# Teamwork and Safety Attitudes in Complex Aortic Surgery at a Dutch Hospital: Cross-Sectional Survey Study

**DOI:** 10.2196/17131

**Published:** 2020-04-08

**Authors:** Alexander D Hilt, Ad A Kaptein, Martin J Schalij, Jan van Schaik

**Affiliations:** 1 Department of Cardiology Leiden University Medical Center Leiden Netherlands; 2 Department of Medical Psychology Leiden University Medical Center Leiden Netherlands; 3 Department of Vascular Surgery Leiden University Medical Center Leiden Netherlands

**Keywords:** human factors, organizational culture, SAQ, SAQ-NL, safety assessment, vascular surgery

## Abstract

**Background:**

Improving teamwork in surgery is a complex goal and difficult to achieve. Human factors questionnaires, such as the Safety Attitudes Questionnaire (SAQ), can help us understand medical teamwork and may assist in achieving this goal.

**Objective:**

This paper aimed to assess local team and safety culture in a cardiovascular surgery setting to understand how purposeful teamwork improvements can be reached.

**Methods:**

Two cardiovascular surgical teams performing complex aortic treatments were assessed: an endovascular-treatment team (ETT) and an open-treatment team (OTT). Both teams answered an online version of the SAQ Dutch Edition (SAQ-NL) consisting of 30 questions related to six different domains of safety: teamwork climate, safety climate, job satisfaction, stress recognition, perceptions of management, and working conditions. In addition, one open-ended question was posed to gain more insight into the completed questionnaires.

**Results:**

The SAQ-NL was completed by all 23 ETT members and all 13 OTT members. Team composition was comparable for both teams: 57% and 62% males, respectively, and 48% and 54% physicians, respectively. All participants worked for 10 years or more in health care. SAQ-NL mean scores were comparable between both teams, with important differences found between the physicians and nonphysicians of the ETT. Nonphysicians were less positive about the safety climate, job satisfaction, and working climate domains than were the physicians (*P*<.05). Additional education on performed procedures, more conjoined team training, as well as a hybrid operating room were suggested by participants as important areas of improvement.

**Conclusions:**

Nonphysicians of a local team performing complex endovascular aortic aneurysm surgery perceived safety climate, job satisfaction, and working conditions less positively than did physicians from the same team. Open-ended questions suggested that this is related to a lack of adequate conjoined training, lack of adequate education, and lack of an adequate operating room. With added open-ended questions, the SAQ-NL appears to be an assessment tool that allows for developing strategies that are instrumental in improving quality of care.

## Introduction

The World Health Organization (WHO) has stated that knowledge on human factors (HF), especially nontechnical skills, is crucial in developing safe environments for patients [1]. A 2017 analysis of the Dutch health care system showed that nontechnical aspects of work were understudied in professional training [2,3]. Nontechnical dimensions of teamwork, such as communication, stress awareness, and shared decision making, all contribute to the effectiveness of teamwork. Importantly, failing to invest in these issues may have negative effects on patient safety and clinical outcomes [4-6]. The challenge lies in how to identify, analyze, and improve these nontechnical skills.

In aviation and offshore industries, for example, awareness of nontechnical skills is crucial in daily work. Training and improving nontechnical skills are often part of corporate policies, with proven effects on safety [7,8]. Similarly, positive results have been observed in health care, although the number of studies is scarce [9,10]. Understanding the safety culture and climate within a team is central to improving nontechnical skills. This can be assessed through questionnaires such as the Safety Attitudes Questionnaire (SAQ), which is a medical HF questionnaire that has been validated in different medical domains. In 2016, the SAQ Dutch Edition (SAQ-NL) was the questionnaire validated in the Dutch language [11,12]. Although often used to assess an *ex ante* baseline and the *ex post* effect of team trainings, the SAQ-NL as a diagnostic tool is not commonly used to identify what exactly needs changing within a team nor to adjust subsequent training accordingly.

The outcome of complex aortic aneurysm surgery is highly dependent on team dynamics. Aortic aneurysms are defined as *complex* when important side branches are included in the aneurysm. This necessitates inclusion of these side branches in the vascular reconstruction, making the procedure high risk. Open, as well as endovascular complex aortic, reconstructions are associated with high mortality and morbidity rates. Both treatments are conducted by multidisciplinary teams.

In this study, the SAQ-NL was used as a diagnostic tool to examine teamwork and safety climate in two types of teams: an open-treatment team (OTT) and an endovascular-treatment team (ETT). The aim of this study was to understand, and ultimately help improve, teamwork conditions and safety climate in this high-risk setting. Primarily, it was hypothesized that (1) the SAQ-NL will provide insight into how teamwork and safety is perceived by different team members and (2) this knowledge may help guide future teamwork improvement strategies.

## Methods

### Terminology

Pinpointing safety culture and safety climate within a medical department is difficult, especially because they are not mutually exclusive. The safety culture of an organization is the product of individual and group values, traditions, perceptions, and competences that determine the commitment to, and the style and proficiency of, an organization’s health and safety management [13]. An organization’s safety culture is the context in which personal safety attitudes develop, persist, and are promoted [8]. It is like a “script” that is taught to every employee that is continuously formed, shaped, and reshaped not only by themselves, but also by their fellow “actors” in the work setting. This concept has been used widely since the 1980s in aviation, as well as industrial settings, such as power plants and offshore environments.

The safety climate is the manifestation of that safety culture in the behaviors and attitudes of professionals, for instance, during surgical procedures. When one would take a “snapshot” of such an environment, certain behavioral cues would be seen; for example, a surgeon being focused on the patient and on his or her tools, the scrub nurse seeing a drop in blood pressure, and the anesthetist reacting accordingly. This “snapshot” with all the interactions between professionals can be seen as the climate people are working in. This climate (ie, the “play” or the day-to-day atmosphere when working) is directly influenced by the department’s culture (ie, the “script” which consists of perceptions, beliefs, and traditions). For example, when convention holds that nurses do not speak up when things go wrong, this negatively impacts the safety climate and often leads to errors and eventually diminished patient safety [14].

**Figure 1:**
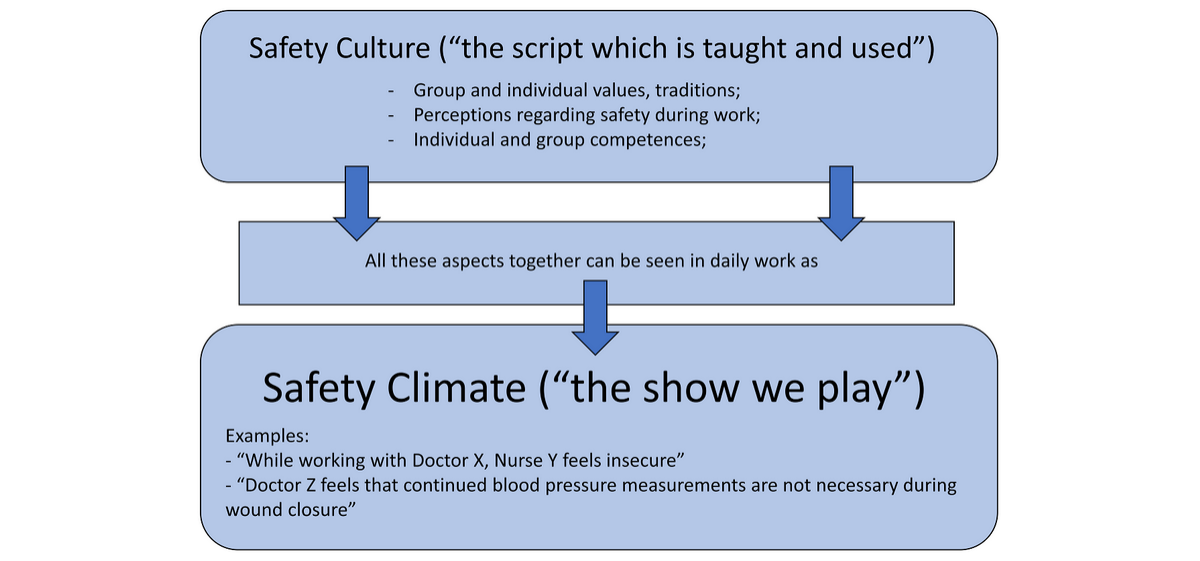
Safety culture and safety climate (source: AD Hilt).

Measuring perceptions of safety and teamwork in a specific setting at a certain point in time (ie, during a surgical procedure) provides insight into the safety climate as well as the safety culture. Put differently, it allows for the assessment of how every “actor” plays their role and, while doing so, to what extent they are influenced by others and the “script” used. Figure 1 gives an overview of the terminology used.

### Design and Study Setting

This study followed a cross-sectional survey design. The Leiden University Medical Centre is one of eight university hospitals in the Netherlands. For this study, two complex aortic aneurysm treatment teams were evaluated: the ETT and the standard OTT.

There were two reasons for the assessment of the two teams. Firstly, the endovascular treatment is relatively new to this hospital, having been performed starting in 2013. Refinement of nontechnical skills is of great interest in this setting, since it has been shown that this improves patient safety and outcomes [10,15]. Secondly, the introduction of the endovascular treatment demanded a shift in work environment for part of the team.

The OTT continued to work in the familiar environment of their operating theater, whereas the ETT had to perform their procedures in an angiography suite, an environment where many team members were not used to working. For daily workflow of the ETT, it was important to understand how it was influenced by this shift in environment. An outline of routine ETT and OTT procedures is shown in Multimedia Appendix 1.

### Study Population

The ETT consisted of 23 team members with a large diversity of radiology personnel, surgical staff, and the addition of a supplier specialist. The OTT consisted of 13 team members with predominantly surgical staff and perfusionists, the latter not being included in the ETT. Noticeably, a supplier specialist was present in the ETT but not the OTT. The specific role of the supplier specialist lies in participating in the discussion of stent type and design, as well as on-site product advice during the procedure. The supplier specialist is a standard, crucial team member of the ETT. Additionally, it should be noted that 2 vascular surgeons, 1 neurologist, and 1 clinical neurophysiology technician were part of both teams. The partial overlap of members of different teams is common in medical settings. All 4 interviewees with dual team membership were able to clearly distinguish between the two teams when answering our questions. In all further analyses, vascular surgeons, thoracic surgeons, radiologists, anesthetists, and neurologists are referred to as *physicians*, whereas scrub nurses, nurse anesthetists, clinical neurophysiology technicians, radiology technicians, supplier specialists, and perfusionists are referred to as *nonphysicians*. Table 1 summarizes the physician and nonphysician composition of both teams, as well as health care tenure and team tenure.

**Table 1:** Overview of team composition in the endovascular-treatment team (ETT) versus the open-treatment team (OTT).

Team and members		N (%)	Average health care tenure, years	Average team tenure, years
**ETT (N=23)**				
	Radiologist	2 (9)	≥10	≥5
	Thoracic surgeon	1 (4)	≥10	4
	Anesthetist	3 (13)	≥10	≥5
	Vascular surgeon	4 (17)	≥10	4
	Neurologist	1 (4)	≥10	3
	Radiology technician	5 (22)	≥10	≥5
	Scrub nurse	3 (13)	8	≥5
	Nurse anesthetist	1 (4)	≥10	≥5
	Clinical neurophysiology technician	2 (9)	≥10	4
	Supplier specialist	1 (4)	8	≥5
**OTT (N=13)**				
	Thoracic surgeon	1 (8)	≥10	3
	Anesthetist	2 (15)	≥10	1
	Vascular surgeon	3 (23)	≥10	≥5
	Neurologist	1 (8)	≥10	4
	Scrub nurse	2 (15)	9	4
	Nurse anesthetist	1 (8)	≥10	4
	Clinical neurophysiology technician	1 (8)	≥10	4
	Perfusionist	2 (15)	≥10	≥5

### Human Factors and the Safety Attitudes Questionnaire

Research into HF aims to understand how humans function in different environments, in order to improve human performance and safety within these environments [16]. HF research has become a core part of major industries, such as aviation and the offshore industry, mainly because of the high dependence on human performance and its effect on safety. Teamwork safety has been extensively evaluated in aviation through HF questionnaires, originally through the Cockpit Management Attitudes Questionnaire (CMAQ) [7,17]. This questionnaire assessed the perceptions concerning safety climate and teamwork among personnel working on an aircraft. This was later refined into the Flight Management Attitudes Questionnaire (FMAQ) [7]. In the medical domain, intensive care units were the first to adopt a medical version of the FMAQ: the Intensive Care Unit Management Attitudes Questionnaire (ICUMAQ) [17]. Developed by Sexton et al, the SAQ is a refinement of the ICUMAQ for a health care setting. It has proven its psychometric and clinical quality in different clinical settings, as well as in the Dutch setting (ie, the SAQ-NL) [11,17,18]. The SAQ assesses 30 items in six domains: safety climate (SC), teamwork climate (TC), job satisfaction (JS), stress recognition (SR), perceptions of management (PoM), and working conditions (WC). The 30 items are each assessed on a 5-point Likert scale: disagree strongly (1), disagree slightly (2), neutral (3), agree slightly (4), and agree strongly (5). The WHO indicates that the SAQ is a valuable HF instrument for assessing medical teamwork dynamics in a standardized fashion [1]. For this study, the strong methodological foundation of the SAQ and its usability in the field were the main reasons to use it.

Additionally, to gain insight into teamwork, safety attitudes, and the meaning of the SAQ-NL outcomes, respondents were asked to answer the following open-ended question: “What are your top three recommendations for improving patient safety in this clinical area?” A Web-based survey of the SAQ-NL via Google Forms (Google) was sent to all ETT and OTT members (see Multimedia Appendix 2).

### Statistics

Frequency tables for gender, professional positions, team tenure, and general health care tenure were generated to give an overview of both teams. Response patterns are shown as percentages. For normally distributed categorical data, a chi-square test was used to calculate statistical differences. For each SAQ dimension, mean scores and standard deviations were calculated per team (ie, ETT and OTT), per professional group (ie, physicians and nonphysicians), and per department. An unpaired *t* test was used to calculate differences between the SAQ-NL mean scores for the ETT and the OTT. A univariate analysis of variance (ANOVA) test was performed to evaluate whether there was a significant difference between average SAQ-NL scores among professional groups, the ETT and OTT, as well as the departments. Data from the open-ended questions were displayed in a descriptive manner; content analysis was used to analyze these. Two authors (ADH and JvS) labelled responses according to major themes that emerged from the data. Cronbach alpha was calculated for all SAQ dimensions of our sample. For analysis, SPSS Statistics for Windows, version 23.0 (IBM Corp), was used. A *P* value of less than .05 was considered significant.

### Biases

Teamwork and safety are delicate subjects, leading to a risk of response bias. Examples of response bias are *question order bias* and *social desirability bias*. The use of a self-administered questionnaire via an online survey is known to minimize the latter effect [19]. All questionnaire data were available only to the main researcher (ADH), who has no professional position in the ETT or the OTT.

### Ethical Considerations

By Dutch law, no ethical approval was needed to conduct this study. All participants gave informed consent for participating in the study and the use of their pseudoanonymized data.

## Results

### Demographics

The ETT consisted of 23 members of which 13 (57%) were male and 11 (48%) were physicians. The OTT consisted of 13 members of which 8 (62%) were male and 7 (54%) were physicians. The composition of the teams regarding number of males and physicians was not significantly different (*P*=.60 and *P*=.50, respectively; see Table 2). Team tenure of 5 years or more was more prevalent among the ETT (12/23, 52%) than among the OTT (3/13, 23%), but this difference was not statistically significant (*P*=.16; see Table 2). Both teams had a large proportion of members working 10 years or more in health care (ETT 19/23, 83%, vs OTT 12/13, 92%, *P*=.30). Long working weeks (ie, ≥50 hours) were more prevalent among the OTT than among the ETT; however, this difference was not significant (OTT 6/13, 46%, vs ETT 5/23, 22%, *P*=.50).

### Mean Scores From the Dutch Safety Attitudes Questionnaire: Endovascular-Treatment Team Versus Open-Treatment Team

An overview of mean SAQ-NL scores with standard deviations per domain is shown in Table 3. Higher means were observed for the OTT; however, an independent-samples *t* test showed that for all SAQ-NL domains, no statistically significant differences existed between the ETT and OTT.

Mean scores for the SAQ dimensions for the ETT and OTT, respectively, were as follows: TC 3.7 (SD 0.37) vs 3.9 (SD 0.31), *P*=.40; SC 3.6 (SD 0.43) vs 3.7 (SD 0.31), *P*=.65; JS 4.1 (SD 0.50) vs 4.2 (SD 0.46), *P*=.39; SR 3.0 (SD 0.73) vs 3.1 (SD 0.92), *P*=.84; PoM 2.9 (SD 0.66) vs 3.1 (SD 0.51), *P*=.44; and WC 3.5 (SD 0.64) vs 3.6 (SD 0.70), *P*=.69. For our sample, all SAQ domains had an acceptable level of reliability (alpha≥.70), with the exception of the TC domain, which had poor reliability (alpha=.58).

**Table 2:** Demographics of the endovascular-treatment team (ETT) and the open-treatment team (OTT).

Demographic	ETT (N=23), N (%)	OTT (N=13), N (%)	*P* value
Male	13 (57)	8 (62)	.60
Physician	11 (48)	7 (54)	.50
Team tenure of ≥5 years	12 (52)	3 (23)	.16
Health care tenure of ≥10 years	19 (83)	12 (92)	.30
Weekly work time of ≥50 hours	5 (22)	6 (46)	.50
Response	23 (100)	13 (100)	N/A^a^

^a^N/A: not applicable.

**Table 3:** Scores from the Safety Attitudes Questionnaire Dutch Edition (SAQ-NL) per domain.

Respondents			Scores for each domain, mean (SD)											
			Teamwork climate		Safety climate		Job satisfaction		Stress recognition		Perceptions of management		Working conditions	
**Team**														
	Endovascular-treatment team (ETT) (N=23)		3.7 (0.37)		3.6 (0.43)		4.1 (0.50)		3.0 (0.73)		2.9 (0.66)		3.5 (0.64)	
	Open-treatment team (OTT) (N=13)		3.9 (0.31)		3.7 (0.31)		4.2 (0.46)		3.1 (0.92)		3.1 (0.51)		3.6 (0.70)	
**Positions within each team**														
	**ETT**													
		Nonphysician^a^ (n=12)		3.6 (0.43)		*3.4 (0.35)^e^*		*3.8 (0.41)^e^*		2.9 (0.61)		2.7 (0.67)		*3.2 (0.68)^e^*
		Physician (n=11)		3.9 (0.31)		*3.9 (0.34)^e^*		*4.4 (0.33)^e^*		3.1 (0.86)		3.1 (0.64)		*3.9 (0.37)^e^*
	**OTT**													
		Nonphysician^a^ (n=6)		3.8 (0.40)		3.7 (0.33)		4.0 (0.47)		3.0 (0.93)		2.9 (0.43)		3.5 (0.54)
		Physician (n=7)		3.9 (0.23)		3.7 (0.33)		4.4 (0.39)		3.1 (0.98)		3.2 (0.52)		3.7 (0.83)
**Department within each team**														
	**ETT**													
		Surgery (n=8)		3.8 (0.35)		3.7 (0.39)		4.0 (0.56)		3.1 (0.62)		2.9 (0.86)		3.3 (0.56)
		Anesthesiology (n=4)		3.9 (0.26)		4.0 (0.32)		4.4 (0.51)		2.6 (1.12)		3.0 (0.00)		4.1 (0.17)
		Radiology (n=7)		3.7 (0.45)		3.4 (0.41)		4.1 (0.46)		3.2 (0.49)		2.5 (0.39)		3.2 (0.79)
		Neurology (n=3)		3.4 (0.20)		3.5 (0.59)		4.0 (0.40)		3.4 (0.76)		3.6 (0.53)		4.0 (0.33)
		Industry (n=1)^b^		4.4		4.1		4.2		2.0		3.6		4.0
	**OTT**													
		Surgery (n=8)		3.9 (0.30)		3.7 (0.38)		4.3 (0.46)		3.0 (1.01)		3.0 (0.51)		3.5 (0.39)
		Anesthesiology (n=3)		3.6 (0.34)		3.7 (0.10)		4.3 (0.61)		2.8 (0.90)		2.8 (0.00)		3.4 (0.96)
		Radiology (n=0)^c^		N/A^d^		N/A		N/A		N/A		N/A		N/A
		Neurology (n=2)		4.0 (0.00)		3.7 (0.40)		4.1 (0.42)		3.8 (0.35)		3.7 (0.42)		4.7 (0.47)
		Industry (n=0)^c^		N/A		N/A		N/A		N/A		N/A		N/A
**Overlapping members within each team**														
	**ETT (n=1 of each)^b^**													
		Vascular surgeon W		4.2		4.2		4.6		2.3		2.4		3.7
		Vascular surgeon X		3.4		3.5		4.2		3.8		2.4		3.4
		Neurologist Y		3.6		4.1		4.4		4.3		3.8		4.3
		Clinical neurophysiology technician Z		3.4		3.3		4.0		3.3		3.0		3.7
	**OTT (n=1 of each)^b^**													
		Vascular surgeon W		4.2		4.2		5.0		1.8		3.4		4.0
		Vascular surgeon X		4.2		3.2		4.4		3.7		2.6		3.3
		Neurologist Y		4.0		4.0		4.4		4.0		4.0		5.0
		Clinical neurophysiology technician Z		4.0		3.4		3.8		3.5		3.4		4.3

^a^Nonphysicians include scrub nurses, nurse anesthetists, clinical neurophysiology technicians, radiology technicians, supplier specialist, and perfusionists.

^b^Because there is only 1 member within this group (or within each group), SDs were not calculated.

^c^Because there are no members in this group, scores were not collected.

^d^N/A: not applicable.

^e^Statistical difference, *P*<.05.

### Mean Scores From the Dutch Safety Attitudes Questionnaire: Physicians Versus Nonphysicians

Univariate ANOVA showed that for the ETT, there were significant differences between physicians and nonphysicians on mean scores for the SC, JS, and WC domains; physicians were significantly more positive about SC, JS, and WC compared to nonphysicians. Mean scores for these domains for physicians versus nonphysicians, respectively, were as follows: SC 3.9 (SD 0.34) vs 3.4 (SD 0.35), *P*=.002; JS 4.4 (SD 0.33) vs 3.8 (SD 0.41), *P*=.001; and WC 3.9 (SD 0.37) vs 3.2 (SD 0.68), *P*=.008. For the ETT, the supplier specialist did not have significantly different scores from the other nonphysicians (see Table 3); there was a slight trend toward higher TC (*P*=.08) and SC (*P*=.07) scores. For the OTT, besides a slight trend toward higher mean scores among physicians for the JS domain—3.7 (SD 0.83) vs 3.5 (SD 0.54), *P*=.12—no significant differences were found between scores from physicians and nonphysicians for all domains.

### Mean Scores From the Dutch Safety Attitudes Questionnaire: Departmental Differences

Univariate ANOVA and independent *t* tests showed no statistical differences between members of different departments (ie, radiology, surgery, neurology, industry, and anesthesiology) among the ETT and OTT.

### Subanalysis of Mean Scores From the Dutch Safety Attitudes Questionnaire: Overlapping Team Members

A total of 3 physicians and 1 technician filled out both the ETT and OTT questionnaires; the mean SAQ-NL scores are also shown in Table 3. An independent *t* test showed no significant differences between the ETT and OTT for any of the SAQ-NL domains in this group. Despite a slight trend toward lower JS among nonphysicians (*P*=.18), no significant differences were found for any of the domains when comparing physicians and nonphysicians in the ETT and OTT, both through univariate ANOVA.

When eliminating these 4 participants from the total analysis of physicians versus nonphysicians in the ETT and OTT, univariate ANOVA showed identical results for the ETT; mean scores for SC (*P*=.002), JS (*P*<.001), and WC (*P*=.008) were significantly lower among nonphysicians compared to physicians in the ETT but not in the OTT.

### Open-Ended Questions

Out of 23 members in the ETT, 21 (91%) respondents together provided 50 comments. Of the 13 members in the OTT, 7 (54%) respondents together provided 14 comments. For the ETT, five themes were identified through content analysis. Comments were related to periprocedural planning; dynamics during procedures, both technical and nontechnical aspects; facilities present in the operating room (OR); and patient privacy (see Multimedia Appendix 3). In total, 23 out of 50 comments (46%) were related to teamwork between nonphysicians and physicians. Nonphysicians expressed their desire to be more involved in the surgical process (12/23 comments, 52%); individual example quotes were as follows: “... more open communication about the patients’ status during surgery,” “... more clarification of the surgical steps taken,” and “... more debriefing after performed surgery.” Physicians found the education of nonphysicians to be an important issue (10/23 comments, 43%); individual example quotes were as follows: “... more time for extra training,” “... more team members should attend the conjoined presurgery meetings,” “... there should be more postsurgery evaluations together,” and “... more open communication at different stages in surgery should be applied toward all.” Additionally, the need for a hybrid OR (ie, fit for both open and endovascular treatment) was stressed (11/50 comments, 22%): “... a hybrid OR where all the radiology and surgery devices are available is a must.”

For the OTT, two major themes were identified; comments were related to periprocedural planning and dynamics during procedures (ie, nontechnical aspects). In total, 6 out of 14 comments (43%) were education related. Nonphysicians wanted to be educated more (4/6 comments, 67%); individual example quotes were as follows: “... there should be more clinical classes about this procedure done by the anesthetist and surgeons” and “... there should be more dedicated trainings and preparation.” Physicians also expressed a desire for more education of nonphysicians in the different phases of surgery (2/6 comments, 33%); individual example quotes were as follows: “... if there are lessons learned during procedures, we should conjointly evaluate them” and “... clinical evaluations after surgery should be evaluated with the whole team.” An overview of relevant themes for both the ETT and OTT with example remarks is included in Multimedia Appendix 3.

## Discussion

### Principal Findings

The results of this study can be summarized as follows: (1) physicians from the ETT were more positive about SC, JS, and WC than were nonphysicians; (2) conjoined training sessions, education, postprocedural evaluation, and a hybrid OR are important topics for future improvements for both physicians and nonphysicians from the ETT; and (3) using the SAQ-NL with the addition of open-ended questions was an instrumental way of assessing the safety culture and climate of two surgical teams and to propose strategies to improve this further.

The findings of our local study suggest that there is room for improvement in teamwork within the ETT. Regarding SC, JS, and WC domains, physicians were more positive than nonphysicians, which was not observed in the OTT. These outcomes were specified by the answers to the open-ended questions. In particular, the remarks regarding more conjoined education on procedures and the desire for a hybrid OR provide a good explanation for the lower scores on the JS and WC domains, and possibly the SC domain, within the nonphysician group. Higher SC, JS, and WC scores reflected aspects of overall perceptions regarding commitment to safety, the work experience, and the quality of the work environment (ie, equipment and staffing), respectively. It is striking that this was different from the OTT. A reasonable explanation for lower JS and WC scores in the ETT may be that nonphysicians need to operate outside of their own habitat, in an environment (ie, the angiography suite) they are not familiar with and do not know as well as the OR. This setup is due to the absence of adequate radiological facilities in the OR. This condition results in nonphysicians having to move large amounts of instruments and materials from the OR to the angiography suite. Having to work outside of their familiar environment and having to move surgical equipment is not necessary for OTT members, who operate in the OR where all materials are close at hand. Qualitative results suggest that building a hybrid OR must be prioritized to raise ETT scores to the level of OTT scores. A hybrid OR is a fully functional surgical theater that is equipped with advanced medical imaging devices, such as fixed C-arms, computed tomography scanners, or magnetic resonance imaging scanners. These imaging devices enable complex, minimally invasive surgery as well as *hybrid* procedures where minimally invasive techniques are combined with conventional *open* surgery.

The perceived need for more education and adequate working conditions could also explain the lower SC score among nonphysicians of the ETT. For future improvements, some suggestions would be cross-functional teaching between radiology technicians and scrub nurses, a more explicit definition of roles and use of equipment, and instruction for team members by physicians. SAQ-NL outcomes can be used after these improvements to measure the effect of these changes in working circumstances on teamwork.

### Implications for Surgical Procedures

Previous studies have shown the effectiveness of using the SAQ as a measure to assess teamwork in different medical settings, largely focusing on measuring the effect of team trainings on daily work [20,21]. The SAQ-NL has not been solely used as a diagnostic tool.

Although no overall differences were found in our study between the ETT and OTT as a whole, there were important differences within the ETT. Physicians were more positive than nonphysicians. Through open-ended questions, important themes for improvement of daily procedures were found. Differences between physicians and nonphysicians are not new [10,22]. However, this is still an important finding, especially for a large tertiary referral hospital. Our findings are not only useful for patient-facing employees, but also for team managers. These findings stress not only the need for facilitating conjoined training and education, but also to direct this more specifically toward the needs of the employees. An example of the latter is *slowing down during surgery*, which enables team members to ask questions at certain key points during the surgical process [23].

### Outcomes of the Dutch Safety Attitudes Questionnaire

Improving health care team culture and teamwork safety is not straightforward, and thorough assessments of workflow and interactions between different professionals are time-consuming. While improvements are necessary, trying to change the entire health care system at once is doomed to fail because of the complex nature of this working environment. For instance, it is questionable what the relevance of a national teamwork assessment would be, essentially assessing teamwork among thousands of people having no direct interaction with each other. Therefore, as proposed by Sexton et al, it is especially important to put effort into the analysis of the working environment of patient-facing employees and focus on local settings [18].

Attitudinal surveys on a local team level can be a valuable addition to this. This study shows that small teams can be fruitfully assessed using the SAQ-NL. Firstly, the strength of using the SAQ-NL among small teams is that a complete response rate is more easily obtained. Secondly, the clinical implications of the study outcomes can be used immediately. For example, regarding the education-related remarks, a focus on more education during procedures can be started during the next surgery. The SAQ-NL could subsequently be used to monitor how such changes would influence a team’s safety attitudes.

Lastly, the SAQ-NL is a useful tool in a cross-professional setting. Due to the intertwinement of work, the supplier specialist, for example, cannot be left out of the ETT analysis. The SAQ-NL in this sense is not restricted to particular professions.

### Future Perspectives: Human Factors and Team Analysis

Assessing team processes such as SC through the SAQ-NL is a valuable addition to team analysis. A recent meta-analysis by Schmutz et al assessed the impact of team process analysis on team performance [24]. It showed that teams who are aware of processes during daily work were almost three times more likely to achieve high performance than teams who were not. In line with this meta-analysis, and as we hypothesized, we recognize the SAQ-NL as a valuable diagnostic tool for team process analysis, mainly to assess and create awareness of processes among team members that define their daily work.

With the knowledge of what needs attention during daily teamwork, a next step could be HF trainings, such as Crew Resource Management (CRM) or Team Strategies and Tools to Enhance Performance and Patient Safety (TeamSTEPPS) [25]. Both are proven to be effective in altering team performance through HF principles. They teach participants that people have certain strengths and weaknesses that can impact daily work in a good or bad way [16,26-28]. The SAQ is often used to monitor the effects of these HF trainings. O’Dea et al proposed in their meta-analysis that, while plausible, it is difficult to unambiguously link changes in team behavior or SAQ outcomes to a particular training [29]. However, regarding the SAQ, starting with a diagnostic approach of what needs attention in a team before commencing training, the effect of CRM or TeamSTEPPS could be better understood during the course of training. For our sample, a CRM or TeamSTEPPS training could aim at improving communication during crucial steps of the ETT procedures, in order to assure shared understanding between physicians and nonphysicians and hereby increase the SC.

### Limitations

Our study has several limitations. Firstly, it is debatable what the clinical meaning or implication is of the difference between sections of the Likert scale in daily work. When looking at the ETT outcomes between nonphysicians and physicians, for example, the difference for the JS domain is 0.6 and for the WC domain is 0.7. What this statistically significant difference implies, solely from the questionnaire’s outcome, is not directly clear. However, using open-ended questions helps us understand this difference. Secondly, we are well aware that there is overlap in respondents filling out the SAQ-NL for both ETT and OTT. In this small group, no differences were found between physicians and nonphysicians for both the ETT and OTT. Correcting all data for this group did not alter the main outcomes. Thirdly, the original SAQ and the SAQ-NL showed good psychometric properties and good reliability (average Cronbach alpha of .76). In our study, the reliability was generally acceptable (alpha≥.70), with the exception of the TC domain, which had rather poor internal reliability (alpha=.58). However, this is highly dependent on the number of subjects participating in the study and the number of items per dimension. Further use of the SAQ-NL and research in this setting should be stressed to evaluate the psychometric properties of the SAQ-NL.

### Conclusions

Nonphysicians of  a local team performing endovascular aortic aneurysm surgery perceived SC, JS, and WC less positively than physicians on the same team. Open-ended questions specified this to be related to a lack of adequate conjoined training, lack of adequate education, and lack of an adequate OR. The SAQ-NL can be a first step in developing strategies to improve quality of care.

## Appendix

Multimedia Appendix 1 Typical endovascular-treatment team (ETT) and open-treatment team (OTT) procedure days.

Multimedia Appendix 2 Safety Attitudes Questionnaire Dutch Edition (SAQ-NL).

Multimedia Appendix 3 Themes and example excerpts from analysis of the Safety Attitudes Questionnaire Dutch Edition (SAQ-NL) for the endovascular-treatment team (ETT) versus the open-treatment team (OTT).

## Abbreviations

ANOVA: analysis of variance
CMAQ: Cockpit Management Attitudes Questionnaire
CRM: Crew Resource Management
ETT: endovascular-treatment team
FMAQ: Flight Management Attitudes Questionnaire
HF: human factors
ICUMAQ: Intensive Care Unit Management Attitudes Questionnaire
JS: job satisfaction
OR: operating room
OTT: open-treatment team
PoM: perceptions of management
SAQ: Safety Attitudes Questionnaire
SAQ-NL: Safety Attitudes Questionnaire Dutch Edition
SC: safety climate
SR: stress recognition
TC: teamwork climate
TeamSTEPPS: Team Strategies and Tools to Enhance Performance and Patient Safety
WC: working conditions
WHO: World Health Organization
