# Supplementary material for: Teamwork and Safety Attitudes in Complex Aortic Surgery at a Dutch Hospital: Cross-Sectional Survey Study
Source: JMIR Hum Factors. 2020 Apr 8;7(2):e17131. doi: 10.2196/17131 (PMC7177441; doi:10.2196/17131)
Supplement: Multimedia Appendix 1 [file humanfactors_v7i2e17131_app1.docx]

# Multimedia appendix 1. Typical ETT and OTT procedure days.

| **ETT procedure day** |  |
| --- | --- |
| 0730hours | Preparation angiosuite   - Radiology material collection from angiosuite supply room (radiology assistant) - Surgery material collection. Moving material from OR to angiosuite (scrub nurse) - Anesthesia material collection. Moving material from OR to angiosuite (anesthesiological team) |
| 0745hours | Technical briefing/material check (interventional radiologist, vascular surgeon, radiology assistant, anesthesiologist, scrub nurse, supplier specialist) |
| 0815hours | Patient briefing in angiosuite (Patient, interventional radiologist, vascular surgeon, radiology assistant, anesthesiologist, scrub nurse, clinical neurophysiologist) |
| 0820hours | Start anesthesia, neuromonitoring preparation (anesthesiologist, clinical neurophysiologist) |
| 0845hours | Surgical preparation   - Radiological material (radiology assistant) - Surgical material (scrub nurse) - Sterile draping (vascular surgeon, scrub nurse, radiology assistant) |
| 0900hours | Surgical cut-down (vascular surgeon, scrub nurse) |
| 0915hours | Start endovascular procedure. Flexible process which might differ greatly from one procedure to another depending on patient, anatomy, stent type, technical/anesthesiological or surgical problems encountered. Usually several ‘stop moments’ or ‘time-outs’ used for team discussion where needed. (Interventional radiologist, vascular surgeon, anesthesiologist, clinical neurophysiologist, supplier specialist, scrub nurse, radiology assistant) |
| …… | Finalization of the procedure |
| 0015hours | Team de-briefing as wounds are closed (whole team) |
| 0030hours | Preparing patient for transport to ICU (anesthesiological team) |
| 0045hours | Transport to, and briefing at ICU (anesthesiologist, vascular surgeon, interventional radiologist) |
| 0100hours | Return of surgery and anesthesia material to OR (scrub nurse, anesthesiological team) |
| **OTT procedure day** |  |
| 0730hours | Preparation operating room   - Surgery material collection on site (scrub nurse) - Anesthesia material collection on site (anesthesiological team) - Perfusion material collection on site (perfusion team) |
| 0800hours | Start anesthesia, neuromonitoring preparation (anesthesiologist, clinical neurophysiologist) |
| 0845hours | Team briefing in OR (thoracic surgeon, vascular surgeon, anesthesiologist, scrub nurses, clinical neurophysiologist, perfusionist) |
| 0900hours | Surgical preparation   - Surgical material (scrub nurse) - Sterile draping (thoracic surgeon, vascular surgeon, scrub nurse) |
| 0915hours | Start surgical procedure. Flexible process which might differ greatly from one procedure to another depending on patient, anatomy, technical/anesthesiologic or surgical problems encountered. Usually several ‘stop moments’ or ‘time-outs’ used for team discussion where needed. (thoracic surgeon, vascular surgeon, anesthesiologist, perfusionist, clinical neurophysiologist, scrub nurse) |
| …… | Finalization of the procedure |
| 0015hours | Team de-briefing as wounds are closed (whole team) |
| 0045hours | Preparing patient for transport to ICU (anesthesiologic team) |
| 0100hours | Transport to, and briefing at ICU (anesthesiologist, thoracic surgeon, vascular surgeon) |

“Angiosuite” = radiology depratment intervention room with primarily radiological equipment, ETT = endovascular treatment team; ICU = intensive care unit; OR = (surgical) operation room; OTT = open treatment team
