# Supplementary material for: Teamwork and Safety Attitudes in Complex Aortic Surgery at a Dutch Hospital: Cross-Sectional Survey Study
Source: JMIR Hum Factors. 2020 Apr 8;7(2):e17131. doi: 10.2196/17131 (PMC7177441; doi:10.2196/17131)
Supplement: Multimedia Appendix 2 [file humanfactors_v7i2e17131_app2.docx]

Multimedia Appendix 2: Safety Attitudes Questionnaire-NL
Below the original English questions are the validated Dutch counterparts. The open question was not in the original SAQ-NL.
 **Teamwork climate**1. Nurse input is well received in this clinical area.

*De inbreng van verpleegkundigen wordt op mijn unit op prijs gesteld.*

2. In this clinical area, it is difficult to speak up if I perceive a problem with patient care.

*Op mijn unit is het moeilijk om het uit te spreken als ik merk dat er een probleem is met de patiëntenzorg.*

3. Disagreements in this clinical area are resolved appropriately (i.e., not *who* is right, but *what* is best for the patient).

*Meningsverschillen op mijn afdeling worden op een goede manier opgelost (d.w.z. niet wie heeft er gelijk,
maar wat is het beste voor de patiënt).*

4. I have the support I need from other personnel to care for patients.

*Ik krijg de ondersteuning die ik nodig heb van staf-artsen om voor patiënten te kunnen zorgen.*

5. It is easy for personnel here to ask questions when there is something that they do not understand.

*Medewerkers op mijn unit kunnen gemakkelijk vragen stellen als er iets is dat ze niet begrijpen.*

6. The physicians and nurses here work together as a well-coordinated team.

*De artsen en de rest van het team hebben hier een goede samenwerking.***Safety Climate**7. I would feel safe being treated here as a patient.

*Als ik hier als patiënt zou worden behandeld, zou ik me veilig voelen.*

8. Medical errors are handled appropriately in this clinical area.

*Medische fouten worden goed afgehandeld op de afdeling.*

9. I know the proper channels to direct questions regarding patient safety in this clinical area.

*Ik weet aan wie ik vragen kan stellen als het gaat om de patiëntveiligheid op de afdeling waar ik werk.*

10. I receive appropriate feedback about my performance.

*Ik krijg goede feedback op mijn functioneren.*

11. In this clinical area, it is difficult to discuss errors.

*Op de unit waar ik werk is het lastig om fouten te bespreken.*

12. I am encouraged by my colleagues to report any patient safety concerns I may have.

*Ik word door mijn collega’s aangemoedigd al mijn bedenkingen wat patiëntveiligheid betreft te melden.*

13. The culture in this clinical area makes it easy to learn from the errors of others.

*De cultuur op mijn unit maakt het makkelijk om van fouten van anderen te leren.*

**Job Satisfaction**14. I like my job.

*Ik ben enthousiast over mijn baan.*

15. Working here is like being part of a large family.

*Het werken in dit ziekenhuis voelt als deel uit maken van een grote familie.*

16. This is a good place to work.

*Dit ziekenhuis is een goede plek om te werken.*

17. I am proud to work in this clinical area.

*Ik ben trots dit ik in dit ziekenhuis werk.*18. Morale in this clinical area is high.

*Het moreel op deze afdeling is hoog.* **Stress Recognition**19. When my workload becomes excessive, my performance is impaired.

*Wanneer mijn werkdruk te hoog wordt, dan lijdt mijn functioneren daaronder.*
20. I am less effective at work when fatigued.

*Als ik vermoeid ben dan verricht ik routinetaken minder goed.*
21. I am more likely to make errors in tense or hostile situations.

*Ik ben meer geneigd om fouten te maken in een gespannen of bedreigende situatie.*

22. Fatigue impairs my performance during emergency situations.

*Vermoeidheid hindert mijn functioneren tijdens acute situaties.*

**Perceptions of management**23. Management supports my daily efforts.

*Het ziekenhuismanagement helpt me bij mijn dagelijkse bezigheden.*
24. Management doesn’t knowingly compromise patient safety.

*Het ziekenhuismanagement brengt de veiligheid van de patiënten niet bewust in gevaar.*
25. Problem personnel are dealt with constructively by our unit / hospital management.

*Dit ziekenhuis gaat constructief om met minder goed functionerend personeel.*
26. I get adequate, timely info about events that might affect my work, from unit / hospital management.

*Ik krijg voldoende, tijdige informatie over gebeurtenissen in het ziekenhuis die invloed kunnen hebben op mijn werk.*

27. The levels of staffing in this clinical area are sufficient to handle the number of patients.

*We hebben genoeg personeel om de werklast aan te kunnen.*

**Working conditions**

28. This hospital does a good job of training new personnel.

*Dit ziekenhuis is goed in het trainen van nieuw personeel.*
29. All the necessary information for diagnostic and therapeutic decisions is routinely available to me.

*Ik beschik steeds over alle informatie die nodig is voor diagnostische en therapeutische beslissingen.*
30. Trainees in my discipline are adequately supervised.

*Degenen die opgeleid worden in mijn discipline krijgen voldoende begeleiding.* **Open question**What are your top three recommendations for improving patient safety in this clinical area? (complex endovascular aortic treatment)
*Wat zijn jouw top drie aanbevelingen om patiëntveiligheid te verbeteren binnen deze setting (complexe endovasculaire aortabehandelingen)*
