# Supplementary material for: Teamwork and Safety Attitudes in Complex Aortic Surgery at a Dutch Hospital: Cross-Sectional Survey Study
Source: JMIR Hum Factors. 2020 Apr 8;7(2):e17131. doi: 10.2196/17131 (PMC7177441; doi:10.2196/17131)
Supplement: Multimedia Appendix 3 [file humanfactors_v7i2e17131_app3.docx]

Multimedia appendix 3: Themes and example excerpts SAQ-NL analysis ETT vs OTT.

| **Teams** | **Example quotes** |
| --- | --- |
| **ETT themes** |  |
| Peri procedural planning | “... sufficient numbers of scrub nurses are needed for safe procedures...” (Scrub nurse) “ ...good preparation of all team members and OR-room…” (Radiology technician) “ ...enough procedures should be planned to keep everyone up-to-date and well-trained...”(Thoracic surgeon) |
| Dynamics during procedure – technical aspects | “ …monitor patient vitals more closely and keep track consistently...”(Scrub nurse) “...radiology technicians and scrub nurses should teach each other the steps they undertake during the procedure…”  (Scrub nurse) |
| Dynamics during procedure – non technical aspects | “…keep track of the steps that are taken during the procedure and discuss what happens next…”(Clinical neurophysiological technician) “…there should be more conjoint post-discussions on the procedure...”  (Vascular surgeon) |
| Facilities of surgical theatre | “...a hybrid OR where all the radiology and surgery devices are available is a must...” (Vascular surgeon, scrub nurse, radiology technician, radiologist) |
| Patient privacy | “...availability of patient data to non-medical personnel/industry is hampered by modern Dutch law...” (Industry proctor) |
| **OTT themes** |  |
| Peri-procedural planning | “… sometimes it’s difficult to plan all the waiting patients...” (Vascular surgeon) “... there should be a limited number of surgeons at the table...” (Perfusionist) “… we should facilitate spectators from other specialities...” (Vascular surgeon) “...we should gather post-operative problems and discuss them more...” (Anesthesist) “...there should be more procedures to keep our skills up-to-date…” (Thoracic surgeon) |
| Dynamics during procedure – non technical aspects | “... there should be a limited number of surgeons at the table to streamline communications...” (Perfusionist) “...education on surgical steps for nurses should be mandatory...” (Scrub nurse) “...ICU personnel should be educated on these procedures as well...”(Vascular surgeon) “…we should do proper introductions of all team members present before the procedure starts...” (Scrub nurse) |

## Definitions of themes:

**Peri procedural planning** - all work involved around the surgical procedure including preparation and discharge of the patient, but also preparation of materials etc.

**Dynamics during procedure** – technical aspects – all steps involved during the procedure that encompass use of equipment (surgical, radiological, neurophysiological) within the OR during the procedure (ETT or OTT).

**Dynamicity during procedure** – non technical aspects – all steps involved during the procedure that encompass communication between team members within the OR during the procedure (ETT or OTT).

**Facilities of surgical theatre** – all aspects of the operating theatre that support functionality (like quick access to equipment) during procedures (ETT or OTT).

**Patient privacy –** all aspects of correct and safe use of patient medical data in line with Dutch law, for example patient rights, safe storage of data and availability of data to third parties.
